# Effectiveness of a Digital Peer-Supported App Intervention in Promoting Smoking Cessations: Nonrandomized Controlled Trial

**DOI:** 10.2196/68638

**Published:** 2025-08-19

**Authors:** Shota Yoshihara, Kayoko Takahashi, Chiaki Uemura, Shin Murakami, Daichi Harada, Hiroshi Yamato

**Affiliations:** 1 Department of Rehabilitation Sciences, Kitasato University Graduate School of Medical Sciences, Kitasato 1-15-1, Sagamihara, Kanagawa, 252-0373, Japan, 81 042 778 8111; 2 A10 Lab Inc, Chuo-ku, Tokyo, Japan; 3 Department of Occupational Therapy, School of Allied Health Science, Kitasato University, Sagamihara, Kanagawa, Japan; 4 Department of Health Development, Institute of Industrial Ecological Sciences, University of Occupational and Environmental Health, Japan, Kitakyushu, Fukuoka, Japan

**Keywords:** smoking, smoking cessation, digital therapeutics, peer support, digital peer support app, mHealth, mobile health, apps

## Abstract

**Background:**

Smoking cessation has become a global priority, with peer support interventions shown to improve abstinence rates. However, no studies have examined the effectiveness of a group-based digital peer-supported app combined with nicotine gum for smoking cessation among working populations.

**Objective:**

This study aimed to assess whether adding a digital peer-supported app to standard nicotine gums improves 12-week smoking abstinence rates among current working smokers in employment-based settings.

**Methods:**

A nonrandomized comparison trial was conducted with current working smokers in Japan. Eligible participants smoked at least 1 cigarette per day, owned a smartphone (iOS or Android), and were enrolled in their company’s health insurance program. Participants were self-selected into one of the two intervention groups (digital peer-supported app + nicotine gums) or a control group (nicotine gums only). The digital peer-supported app creates a group chat for up to 5 people aimed at smoking cessation, where participants can anonymously post counts, photos, and comments daily. Logistic regression analyses adjusted for demographic and smoking-related variables were used to estimate the odds ratios for smoking cessation. Engagement with the app (usage days and posting frequency) was analyzed within the intervention groups.

**Results:**

A total of 451 participants were included in the per-protocol analysis (260 in the intervention groups and 191 in the control group). The 12-week abstinence rate was significantly higher in the digital peer-supported app + nicotine gum group compared to the gum-only group (59.2% [154/260] vs 38.7% [74/191]). The adjusted odds ratio of smoking cessation was 2.41 (95% CI 2.07‐2.81), indicating a significant impact of digital peer support. Both higher duration of digital peer-supported app usage and increased posting frequency were positively associated with cessation success (*P* for trend <.001).

**Conclusions:**

The addition of a digital peer-supported app to nicotine gum use significantly improved smoking cessation outcomes among working smokers. These findings provide preliminary evidence for the feasibility and effectiveness of integrating group-based digital peer support into smoking cessation interventions.

## Introduction

Smoking is the leading cause of noncommunicable disease mortality in Japan, significantly increasing the risk of ischemic heart disease, stroke, chronic obstructive pulmonary disease, and cancer [1-3]. The societal burden of smoking has intensified, marking smoking cessation as a global imperative [4].

Various measures to promote smoking cessation have been implemented in Japan, including a raised tobacco price (taxation: 59.9%) [5] and enforcement of the revised Health Promotion Act that prohibits smoking in public facilities other than designated smoking areas [6]. Under the Japanese national insurance system, patients with nicotine dependence are eligible for covered smoking cessation treatment. This program lasts 12 weeks and includes 5 visits with health care professionals, during which participants receive counseling and pharmacotherapy treatments, such as nicotine patches and gums. Despite these efforts, smoking cessation success rates in Japan remain modest. According to a report by the Ministry of Health, Labour and Welfare, the average completion rate for the standard 5-session smoking cessation program was 34.5%, with hospitals averaging 43.5% and clinics averaging 32.7% [7].

Peer support–based interventions, which have become a common approach for promoting health-related behavior change [8], have been defined as “a method of teaching or facilitating health promotion that makes use of people sharing specific health messages with members of their own community” [9]. A meta-analysis suggests that interventions involving peer support may significantly increase smoking abstinence rates [10]. Additionally, digital therapies, including smartphone apps, have shown promise. Systematic reviews have confirmed the effectiveness of smoking cessation apps [11,12]. A digital peer-supported app, which facilitates group interactions to motivate users toward quitting smoking, could be particularly beneficial [13-15]. However, no studies have specifically evaluated the effectiveness of this digital peer-supported app for smoking cessation. Investigating the potential benefits of integrating the digital peer-supported app with behavioral support could provide valuable insights.

This study aims to investigate whether adding a digital peer-supported app to nicotine gums can improve the 12-week smoking abstinence rate among current working smokers.

## Methods

### Study Design

A web-based questionnaire was administered to employees from 3 companies (electronics, insurance, and telecommunications industries) affiliated with a health insurance association from October 2022 to February 2023. This nonrandomized comparison trial assessed the effectiveness of a digital peer-supported app in promoting smoking cessation among current working smokers. Smoking cessation success rates were then compared, with the group using only the nicotine gums serving as the reference.

### Ethical Considerations

This study received ethical approval from the Research Ethics Committee of the Institutional Review Board of the School of Allied Health Sciences at Kitasato University (approval number: 2024‐008). At the time of enrollment, participants were also informed that data obtained through their involvement in the program might be used for research purposes in an anonymized form, and participation was contingent upon their agreement to these terms. An opt-out method was used to obtain informed consent, with study details publicly disclosed on the web. Participants could withdraw at any time. No financial reward was offered. All data were anonymized to ensure privacy.

### Participants

This study enrolled employed individuals who were current smokers. Eligibility criteria included (1) ownership of a smartphone compatible with iOS or Android operating systems, (2) enrollment in their company’s corporate health insurance program, and (3) self-reported daily smoking of at least 1 cigarette.

Participants self-selected into one of the two intervention groups: (1) nicotine gum only (Nicorette 2 mg) or (2) a combination of the digital peer-supported app and nicotine gum. Participation in the app-based intervention was optional.

### Smoking Cessation Program

**Figure 1.**
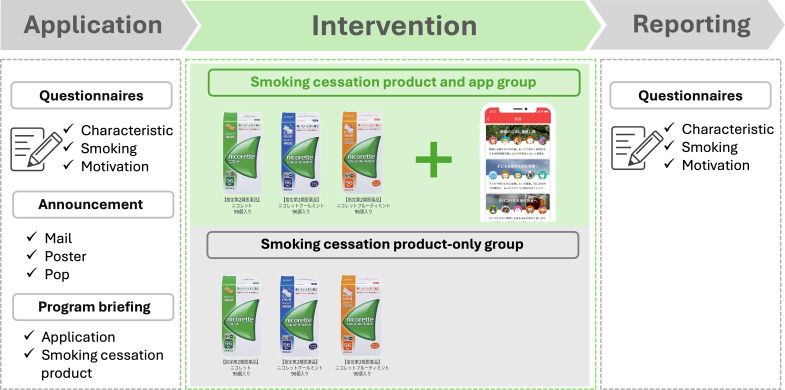
Timeline of study procedures.

The smoking cessation program was conducted remotely and lasted 12 weeks (Figure 1). Participant recruitment took place over approximately 20 days, beginning about 1 month before the program’s start. Recruitment strategies included both individual outreach and workplace-based promotion. Potential participants were primarily invited via email communications sent by their employers and affiliated health insurance societies. In addition, promotional materials—such as posters and triangular pop-up displays—were distributed and displayed at the participants’ workplaces to raise awareness of the program. Interested individuals voluntarily enrolled in the program by independently submitting an application form, typically via a web-based platform.

A preprogram online session was held to explain the program’s structure and educational content, which was designed to assist participants in quitting smoking. This content included discussions on withdrawal symptoms and how to use the digital peer-supported app effectively. Educational videos featured an ex-smoking physician discussing the health impacts of smoking, the benefits of quitting, coping strategies for withdrawal, methods for creating a smoke-free environment, and planning alternative behaviors.

For those unable to attend the live session, an e-learning course was available. The program also offered participants nicotine gums (96 pieces per box) without a prescription. An instructional video on how to use the smoking cessation aids was provided to all participants before the challenge began.

### The Digital Peer-Supported App Intervention

The digital peer-supported app “Minchalle” is a smartphone app developed by A10 Lab Inc, which was initially released in November 2015 and is available for Android and iPhone [13-15]. This app was originally designed to help users build desirable habits, such as exercising, dieting, and learning English conversation, and has been downloaded over 1.6 million times. For this study, the app was adapted to support smoking cessation.

**Figure 2.**
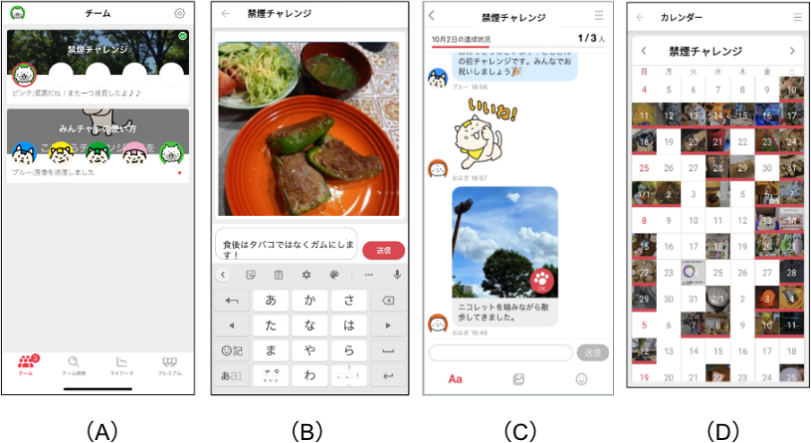
Examples of app screens. (A) Select a group of 5 members. (B) Upload a photo of the day with a comment related to abstinence. (C) Read responses and applause from the other 4 members of the group. (D) Look back at the records of abstinence by photos.

In this smoking cessation program, participants are enrolled by registering and installing the app. They were automatically assigned to teams of up to 5 individuals based on the order of registration. Team members interacted anonymously using nicknames and engaged in group chats, where they could post photos, messages, and stamps to support one other (Figure 2). To ensure privacy, the app was designed to prevent the sharing of personally identifiable information, and participants were instructed not to include such information in their posts.

Two types of photo-posting features were available: Challenge Photo and Normal Photo. Challenge Photo allowed users to post 1 photo with a comment per day to report their progress in quitting smoking. These were recorded in a personal calendar view (Figure 2), supporting self-monitoring and reflection. Normal Photo allowed users to post additional photos without comments, typically after submitting a Challenge Photo for the day, enabling more spontaneous sharing within the team.

Additional key features included (1) peer approval of posts, (2) automated feedback based on team engagement, and (3) automatic removal from the group if a participant exceeded a specified inactivity period of 15 consecutive days. Although participants can post comments or photos more than once a day and interact with other 4 members, such engagement is not mandatory. The digital peer-supported app is available at no cost to the participants. For visual representation, examples of the user interface can be found in Multimedia Appendix 1.

### Other Interventions

Throughout the smoking cessation program, participants received supplementary support via a chat platform. This support included guidance on managing withdrawal symptoms in daily life, quizzes designed to educate participants about the adverse health effects of smoking, and information on the physiological changes associated with quitting smoking. Additionally, a consultation service was available, allowing participants to receive personalized responses from medical professionals. Participants could submit individual concerns, such as withdrawal symptoms, proper use of cessation aids, or other issues encountered during the program, through a web-based consultation form. Inquiries were also accepted via email. Medical professionals responded to each consultation individually, typically within 1 week of submission.

### Measurements

#### Baseline Characteristics

Baseline demographic variables, including age and sex, and smoking-related characteristics—such as motivations for quitting, smoking duration, and daily cigarette consumption—were obtained through a web-based survey. These variables were originally collected using predefined categories in the survey instrument. Age was grouped as 20‐29, 30‐39, 40‐49, 50‐59, and 60 years or older; sex was categorized as male or female. Smoking duration was classified as less than 5, 5‐9, 10‐19, 20‐29, and 30 years or more. Daily cigarette consumption was recorded in categories: 1‐9, 10‐19, and 20 or more cigarettes per day. The purpose of smoking cessation was assessed using a multiple-choice format (eg, my health, saving for money, family’s health, none, or others). Motivation for smoking cessation was measured on a scale of 1 to 10, with higher scores indicating stronger motivation to quit smoking.

#### Log Data of the Digital Peer-Supported App

The smoking cessation program was conducted remotely over a 12-week period. Participants were recruited over a 20-day period beginning approximately 1 month prior to the program’s start, and they had access to the digital peer-supported app for the full duration of the intervention.

To evaluate participants’ engagement with the app, we collected log data automatically recorded by the system. Two behavioral indicators were analyzed: (1) continuous app usage and (2) posting frequency during the 12-week program. Continuous app usage was measured by counting the number of days on which participants logged into the app. Posting frequency was defined as the total number of posts (including messages, challenge posts, normal photos, and stamps) made by each participant throughout the program.

For both indicators, participants were classified into 3 groups using tertile cutoffs: low, moderate, and high. These categories were used in the analysis to explore the association between different levels of engagement and smoking cessation outcomes.

### Outcome

In this program, continuous smoking cessation is defined as self-reported abstinence from smoking for a minimum of 4 weeks. Individuals are classified as “quitters” if they report having abstained from smoking entirely, without consuming a single cigarette, for at least 4 weeks by the end of the program. This information is collected via a web-based form on the program’s final day, with cessation determined by the “last smoking date” provided by participants.

### Statistical Analysis

A logistic regression analysis was conducted to estimate the odds of smoking cessation. The outcome was treated as a binary variable (1=quit, 0=not quit). The nicotine gum–only group served as the reference, and the odds ratio (OR) for quitting was calculated for the group using both the app and nicotine gum.

The analysis included statistical adjustments made in multiple steps: model 1 adjusted for age (categorized as 20‐29, 30‐39, 40‐49, 50‐59, and 60 years or older), sex (male or female), and cluster (3 study sites). Model 2 incorporated additional adjustments for the duration of smoking (categorized as 0‐4, 5‐9, 10‐19, 20‐29, and 30 years or more) and daily cigarette consumption (categorized as 1‐9, 10‐19, and 20 cigarettes or more). Model 3 was further adjusted for the purpose of smoking cessation (my health, saving for money, family’s health, none, or others) and motivation for smoking cessation (treated as a continuous variable). To account for potential clustering effects within the company, we used robust standard errors clustered by company.

Furthermore, logistic regression was used to evaluate the impact of digital peer-supported app usage duration and posting frequency on smoking cessation success, specifically among participants who used the app. The “low” tertile group was used as the reference category. To assess linear trends across tertiles, *P* values for trend were calculated using orthogonal polynomial contrasts (specifically, the “contrast” command in Stata), with the same covariate adjustments as in model 3.

All statistical analyses were conducted using Stata, version 17.0 (Stata Corp), with statistical significance defined as a 2-tailed *P* value of <0.05. The OR and the corresponding 95% CI were calculated to assess the strength of associations between variables.

## Results

### Participants

Initially, 542 participants enrolled in the smoking cessation program. Among them, 90 (16.6%) participants did not use the nicotine gum at all during the program, and 1 participant had missing age data. These individuals were excluded from the analysis.

As a result, the final per-protocol analysis set included 451 participants: 260 participants used both the digital peer-supported app and nicotine gums, while 191 used only nicotine gums. Most participants were male (434/451, 96.2%), 61.6% (278/451) were over the age of 50 years, and 68.7% (310/451) reported a smoking history of 20 years or more.

### Participant Characteristics

Table 1 presents the baseline characteristics of the participants. Compared to the group using only the nicotine gums, participants using both the digital peer-supported app and the nicotine gums had a higher average age, a longer history of smoking, and were more likely to cite “family health” as their primary motivation for quitting.

**Table 1.** Participants’ characteristics at baseline.

	Total (n=451)	Nicotine gums–only group (n=191)	Digital peer-supported app and nicotine gums (n=260)
Sex, n (%)			
Male	434 (96.2)	190 (99.5)	244 (93.8)
Female	17 (3.8)	1 (0.5)	16 (6.2)
Age (years), n (%)			
20‐29	6 (1.3)	4 (2.1)	2 (0.8)
30‐39	70 (15.5)	39 (20.4)	31 (11.9)
40‐49	97 (21.5)	45 (23.6)	52 (20)
50‐59	171 (37.9)	68 (35.6)	103 (39.6)
60＋	107 (23.7)	35 (18.3)	72 (27.7)
Duration of smoking (years), n (%)			
0‐4	14 (3.1)	8 (4.2)	6 (2.3)
5‐9	37 (8.2)	20 (10.5)	17 (6.5)
10‐19	90 (20)	44 (23)	46 (17.7)
20‐29	167 (37)	65 (34)	102 (39.2)
30＋	143 (31.7)	54 (28.3)	89 (34.2)
Cigarettes/day, n (%)			
1‐9	72 (16)	29 (15.2)	43 (16.5)
10‐19	299 (66.3)	126 (66)	173 (66.5)
20＋	80 (17.7)	36 (18.8)	44 (16.9)
Purpose of smoking cessation, n (%)			
My health	250 (55.4)	113 (59.2)	137 (52.7)
Saving for money	129 (28.6)	51 (26.7)	78 (30)
Family’s health	46 (10.2)	12 (6.3)	34 (13.1)
None	14 (3.1)	8 (4.2)	6 (2.3)
Others	12 (2.7)	7 (3.7)	5 (1.9)
Motivation for smoking cessation, mean (SD)	7.9 (2.1)	7.7 (2.2)	8.1 (2)

### Digital Intervention and Smoking Abstinence Rates

Logistic regression analyses demonstrated that participants using the digital peer-supported app in combination with nicotine gums had significantly higher smoking abstinence rates at 12 weeks compared to those using only the nicotine gums (59.2% [154/260] vs 38.7% [74/191]), as shown in Table 2. Model 1 yielded an OR of 2.40 (95% CI 2.02‐2.85), model 2 reported an OR of 2.41 (95% CI 2‐2.9), and model 3 showed an OR of 2.41 (95% CI 2.07‐2.81), indicating a statistically significant improvement associated with the digital intervention.

**Table 2.** The results of the logistic regression analyses on smoking cessation success ratios.

	Nicotine gums–only group	App and nicotine gums group
Participants, n	191	260
Prevalent cases, n (%)	74 (38.7)	154 (59.2)
Model 1,^a^^,^^b^ OR^c^ (95% CI)	1.00 (reference)	2.40 (2.02‐2.85)
Model 2,^a^^,^^d^ OR (95% CI)	1.00 (reference)	2.41 (2.00‐2.90)
Model 3,^a^^,^^e^ OR (95% CI)	1.00 (reference)	2.41 (2.07‐2.81)

a In all models, companies were incorporated as clusters.

b Adjusted for age (in years, continuous) and sex (male or female).

c OR: odds ratio.

d Adjusted for age (in years, continuous), sex (male or female), duration of smoking (0‐4, 5‐9, 10‐19, 20‐29, or 30 years or more), and the number of cigarettes smoked per day (1‐9, 10‐19, or 20 cigarettes or more).

e Adjusted for age (in years, continuous), sex (male or female), duration of smoking (0‐4, 5‐9, 10‐19, 20‐29, or 30 years or more), the number of cigarettes smoked per day (1‐9, 10‐19, or 20 cigarettes or more), purpose of smoking cessation (my health, saving for money, family’s health, none, or others), and motivation of smoking cessation (continues).

### Smoking Cessation Success and Digital Peer-Supported App Engagement

The relationship between smoking cessation success rates and both the duration of digital peer-supported app usage and posting frequency, restricted to participants in the digital peer-supported app group, is presented in Tables3  4. The analysis restricted to participants using the digital peer-supported app showed that those in the moderate and high tertiles for digital peer-supported app usage duration and posting frequency had significantly higher smoking cessation success rates compared to those in the low tertile (*P* for trend <.001).

**Table 3.** Association between the duration of app usage and smoking cessation success ratios among users of digital peer-supported app and nicotine gums.

	The duration of app usage			
	Low (0‐62 days)	Moderate (63‐106 days)	High (107‐136 days)	*P* for trend
Participants, n	95	85	80	
Prevalent cases, n (%)	43 (45.3)	54 (63.5)	57 (71.3)	
Model 3,^a^ OR^b^ (95% CI)	1.00 (reference)	1.39 (1.31‐1.48)	3.43 (3.11‐3.79)	<.001

a Adjusted for age (in years, continuous), sex (male or female), duration of smoking (0‐4, 5‐9, 10‐19, 20‐29, or 30 years or more), the number of cigarettes smoked per day (1‐9, 10‐19, or 20 cigarettes or more), purpose of smoking cessation (my health, saving for money, family’s health, none, or others), and motivation of smoking cessation (continues). In model 3, companies were incorporated as clusters.

b OR: odds ratio.

**Table 4.** Association between posting frequency and smoking cessation success ratios among users of a digital peer-supported app and nicotine gums.

	Posting frequency			
	Low (0‐5 times)	Moderate (6‐24 times)	High (25‐449 times)	*P* for trend
Participants, n	90	84	86	
Prevalent cases, n (%)	39 (43.3)	48 (57.1)	67 (77.9)	
Model 3,^a^ OR^b^ (95% CI)	1.00 (reference)	1.16 (0.88‐1.53)	3.67 (3.36‐4.02)	<.001

a Adjusted for age (in years, continuous), sex (male or female), duration of smoking (0‐4, 5‐9, 10‐19, 20‐29, or 30 years or more), the number of cigarettes smoked per day (1‐9, 10‐19, or 20 cigarettes or more), purpose of smoking cessation (my health, saving for money, family’s health, none, or others), and motivation of smoking cessation (continues). In model 3, companies were incorporated as clusters.

b OR: odds ratio.

No adverse events directly attributable to the digital peer-supported app were reported.

## Discussion

### Principal Results

This study assessed the effectiveness of a digital peer-supported app for smoking cessation within an online group setting, marking a novel therapeutic approach. We observed a significant increase in smoking abstinence rates among patients with nicotine dependence who used the digital peer-supported app alongside standard cessation products. To our knowledge, this is the first study to evaluate the efficacy of a group-based, digital peer-supported app in improving smoking abstinence rates. This also highlights the feasibility of incorporating such digital tools in smoking cessation interventions and contributes preliminary evidence to support the development of digital health solutions.

### Comparison With Previous Studies

Our findings are notably significant. The primary outcome—self-reported smoking abstinence at week 12—was substantially high in the group using both the digital peer-supported app and nicotine gums (154/260, 59.2%), which was higher compared to the group using only the nicotine gums (74/191, 38.7%). Our results coincide with previous research on digital smoking cessation programs using nongroup-based, single-user apps, which typically show success rates about 5% to 15% higher [16-19]. Our findings suggest that a group-type digital peer-supported app, when combined with nicotine gum, may enhance short-term smoking cessation outcomes compared to nicotine gum alone. However, the results should be interpreted with caution due to the reliance on self-reported data and the lack of long-term follow-up.

Our study suggests that a digital peer-supported app, informed by social cognitive theory [20], can significantly aid smoking cessation. To further substantiate these findings, we focused on participants who actively engaged with the digital peer-supported app, examining the relationship between smoking cessation success rates, duration of digital peer-supported app use, and posting frequency. The analysis indicated that the duration of use or posting frequency was significantly greater in the groups with moderate and high cessation success rates compared to the groups with lower success rates. A plausible explanation for this is that individuals who engaged with the digital peer-supported app more extensively and frequently posted updates had increased opportunities to share their success stories, receive recognition, and obtain positive feedback through the chat feature. This likely enhanced their motivation and self-efficacy [21], a phenomenon similar to what has been observed in traditional face-to-face peer support studies [22].

The digital peer-supported app is designed to integrate seamlessly into the daily communication environments of smokers, offering support that is more accessible than traditional methods such as group sessions, telephone calls, or websites. The digital peer-supported app accessibility has likely contributed to increased engagement with quitting efforts [23,24]. In addition, while the app did not have any dedicated features for tracking or managing nicotine gum use, the digital peer support it facilitated may have indirectly encouraged better adherence. This enhanced adherence is one possible factor that might have contributed to the higher smoking cessation success rates observed in the intervention group. We posit that the digital peer-supported app may have mirrored traditional peer-support dynamics, where individuals are known to quit smoking in interconnected groups [25].

### Limitations and Future Study

This study has several limitations. First, smoking cessation outcomes were assessed through self-reported data without biochemical validation (eg, carbon monoxide or cotinine testing) [26], which may overestimate true smoking cessation rates. Second, the nonrandomized design and participant self-selection into intervention groups may have introduced bias, thereby limiting the ability to draw causal inferences. Third, the study targeted individuals with smartphone access, potentially introducing selection bias, as more motivated or tech-savvy individuals may have been more likely to enroll. Additionally, the inclusion criterion of smoking at least 1 cigarette per day may limit generalizability to heavy smokers. Fourth, the study did not assess long-term cessation beyond the 12-week intervention, leaving the sustainability of effects unclear [27,28]. Fifth, while participants used anonymous nicknames, some may have known each other due to recruitment from the same or affiliated companies, potentially influencing team dynamics. Sixth, although a consultation service with medical professionals was available throughout the program, its potential impact on smoking cessation could not be evaluated. Only 13 consultation requests were submitted during the intervention period, and all entries were anonymized, making it impossible to identify which participants used the service or to assess its association with cessation outcomes. This introduces a possible confounding factor that could not be statistically controlled. Seventh, nicotine dependence was not formally assessed using standardized assessments (eg, Fagerström test) [29-31], limiting our understanding of baseline addiction severity [32]. Eighth, the use of heated tobacco products and e-cigarettes was not assessed, which may have influenced smoking behaviors and cessation outcomes [33,34]. Ninth, although some participants mentioned withdrawal-like symptoms in group chats, no standardized assessment of withdrawal symptoms was conducted. Nicotine gum usage was self-managed and not tracked; all participants received 2 mg gum (96 pieces) intended for the 12-week program, with no additional supply provided, and no choice of higher dose. The uniform dosing may have been insufficient for heavier smokers. Future studies should consider tailoring nicotine replacement therapy based on individual smoking behaviors. Tenth, no formal sample size calculation was performed due to the lack of previous studies on this specific digital peer support smoking cessation program. Finally, the participants in this study consisted primarily of male employees from large Japanese companies, limiting generalizability to other populations, including women and nonworking individuals. Future studies should explore the sustainability of these outcomes and assess whether prolonged digital peer-supported app engagement or follow-up support is necessary to maintain smoking cessation.

### Conclusions

Our findings suggest that using a digital peer-supported app may boost smoking abstinence rates over 12 weeks in current working smokers, compared to using standard nicotine gums alone.

## Supplementary material

10.2196/68638 Multimedia Appendix 1 Examples of the user interface.

## Abbreviations

OR: odds ratio
